# STED-Inspired Cationic Photoinhibition Lithography

**DOI:** 10.1021/acs.jpcc.3c04394

**Published:** 2023-09-07

**Authors:** Sourav Islam, Marco Sangermano, Thomas A. Klar

**Affiliations:** † Institute of Applied Physics, Johannes Kepler University Linz, 4040 Linz, Austria; ‡ Department of Applied Science and Technology, Politecnico Di Torino, 10124 Torino, Italy

## Abstract

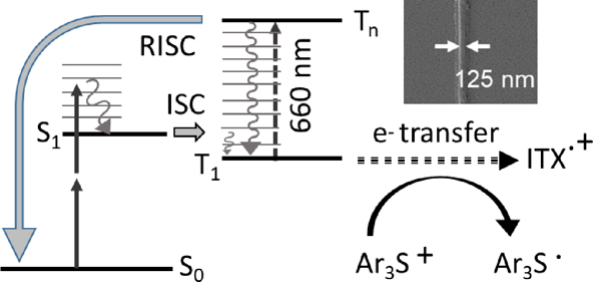

Direct laser writing by two-photon lithography has been enhanced substantially during the past two decades by techniques borrowed from stimulated emission depletion (STED) microscopy. However, STED-inspired lithography was so far limited to radical polymerizations, mostly to acrylates and methacrylates. Cationic polymers did not derive benefits from this technique. Specifically, epoxide polymerization, which plays a paramount role in semiconductor clean-room technology, has not yet been reported with a second, depleting laser focus in the outer rim of the point spread function. We now found that using a thioxanthone as a sensitizer and sulfonium or iodonium salts as photoinitiators enables at least partial optical on/off switching of two-photon polymerization and, in the case of the sulfonium salt, allows for writing epoxy lines with widths shrunk by approx. two-thirds compared to lines written with two-photon polymerization alone.

## Introduction

STED-inspired lithography can be used to improve the feature widths and resolutions of multiphoton lithography (MPL), where multiple photons of a visible or near-infrared short-pulse laser are used to excite the photoinitiators.^1^ Compared to standard UV-based lithography, MPL has two decisive advantages: First, it bears an intrinsic three dimensionality, and second, it uses less photon energy compared to mid- to far-UV or e-beam lithography. Unfortunately, the larger wavelength poses restrictions to the minimal feature size, being about 100 nm in the state-of-the-art acrylate-based MPL.^2,3^

In the 90s, it has been proposed^4^ and experimentally shown^5^ that diffraction-unlimited fluorescence microscopy is possible using stimulated emission depletion (STED) in the outer rim of the focal point spread function (PSF). Soon, it became clear that not only STED but any technique that reversibly switches off excited fluorophores by a second laser beam can in principle be used for far-field imaging beyond the diffraction limit.^6^ Inspired by this success in microscopy, optical switching in the outer rim of the PSF has been applied to nanometrically control photoinitiators of radical polymerization reactions, and hence, structure sizes down to the deep-subwavelength scale could be achieved.^7−11^ These and many other reports on STED-inspired photoinhibition lithography have in common that they focus on radical polymerizations, mostly of (meth)acrylates.^12^ Only a few records on STED-inspired lithography beyond radical polymerization exist. For instance, individual chemical click reactions can be used, where a molecule can either be reversibly switched into a reactive isomer with one wavelength and switched back to an unreactive one with another wavelength of light,^13,14^ or where individual redox reactions can be optically activated with one wavelength and prevented with another one.^15^ These are individual reactions, not polymerizations; however, they can be used in a photoresist by inducing gelation.^16,17^ Importantly, the relevant class of cationic (and also anionic) polymerizations eluded STED-inspired photoinhibition lithography, so far. This is all the worse as epoxy-based resins are generally less toxic and show reduced shrinkage compared to acrylates and they are the workhorse of semiconductor clean-room lithography.^18^ Notably, using MPL alone to polymerize epoxides, the linewidths are typically four to five times as thick as comparable linewidths achieved with (meth)acrylates.^19,20^ Consequently, the minimal feature sizes achievable by MPL are more in the range of half a micrometer in the case of epoxides, instead of 100 nm as it was achieved with acrylates.^2^ From this starting point, the applicability of STED-inspired photoinhibition lithography to cationic photopolymerization would be highly welcome.

**Figure 1:**
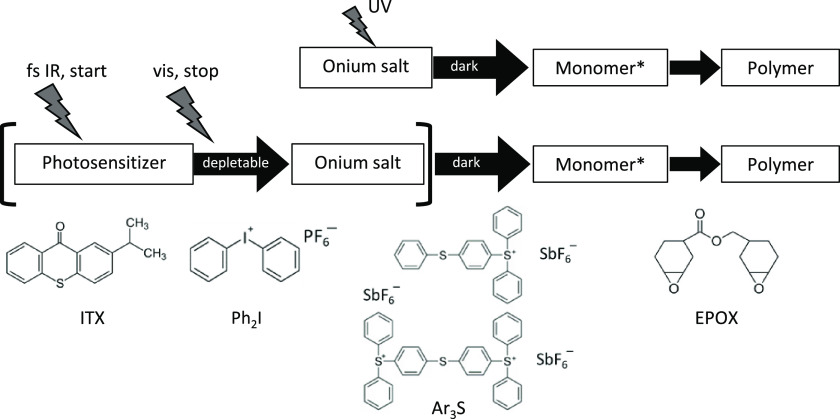
Upper line: usually, an onium salt is excited by UV light and activates a monomer, which then polymerizes. The process is fast and comprises optically inaccessible (dark) reactions. Middle line: a photosensitizer, excitable with near-UV or the corresponding femtosecond near-IR pulses, sensitizes the onium salt. This process is slow and can be stopped (depleted) by visible light. Lower line: structures of the used chemicals: ITX as a photosensitizer, either Ph_2_I:PF_6_ or Ar_3_S:SbF_6_ as onium salts, and EPOX as an epoxy monomer.

The upper line in Figure 1 shows a common strategy to photopolymerize epoxy resins. The polymerization of the monomers is optically started via an onium salt.^21,22^ These photoinitiators typically show strong absorbances in the UV range below 300 nm (iodonium salts)^21^ or below 350 nm (sulfonium salts)^22^ but no absorption above these wavelengths. Rapidly after excitation, they initiate the polymerization via the photogenerated strong Brønsted acid through a cationic chain growth process. These facts make this classical strategy useless for our purpose for two reasons: First, we are aiming for a resist that is excitable in the very near UV (to fit a two-photon excitation by 780 nm femtosecond pulses), and second, the reaction steps after photoexcitation of the onium are “dark” in the sense that they cannot be manipulated optically anymore after they have been triggered by light. Hence, the reaction cannot be stopped by a second laser beam, and STED-inspired lithography will not work. The first issue has been addressed by using photosensitizers absorbing in the near-UV to blue spectral range,^23^ specifically by using thioxanthones including isopropyl thioxanthone (ITX).^22,23^ Boiko et al. have shown that a sensitizer/initiator doublet comprising a thioxanthone and an onium salt can also be used for MPL using a Ti:Sapphire femtosecond laser.^19^ We sketch this approach in the middle line of Figure 1. Importantly, ITX is well-known to be a photoswitchable initiator, useful for STED-inspired acrylate lithography.^9^ ITX proved to be optically depletable via transient-state absorption when using wavelengths from 530 to 700 nm.^24−26^ In the following, we will abbreviate this process with TAD: “transient-state absorption depletion”. Several other thioxanthones have been screened for STED-inspired radically polymerizing lithography; however, ITX proved to be the most effective.^27,28^ Further, a large body of work, both theoretical and experimental, is available on the photophysics of thioxanthones.^29−40^

Here, we report, to the best of our knowledge for the first time, about a successful attempt to nanostructure an epoxy resist by means of STED-inspired photoinhibition lithography and show that lines written with photoinhibition show an approx. 66% smaller width compared to lines written with sheer two-photon lithography. Using published numerical values of the dynamics of ITX and similar thioxanthones’ triplet states and the dynamics of photoinitiation of onium salts by ITX, we are able to give an explanation for the process of photodepletion.

## Materials and Methods

We used a homebuilt dual-beam two-photon lithography setup similar to the one described before.^11^ The photoresists used for the experiments were a mixture of 3,4-epoxycyclohexylmethyl 3,4-epoxycyclohexanecarboxylate (EPOX, Sigma Aldrich) as a monomer, triarylsulfonium hexafluoroantimonate of 50 wt % in propylene carbonate (AR_3_S:SbF_6_, Sigma Aldrich, prod. no. 654027) or diphenyliodonium-hexafluorophosphate (Ph_2_I:PF_6_, Sigma Aldrich, prod. no. 548014) as onium salts, and 2-isopropylthioxanthone (ITX, Sigma Aldrich) as a photosensitizer. Several different mixtures of ITX and onium salts were investigated to find the best composition for nanofabrication (see Supporting Information, Figures S1 and S2). For both onium salts, the best results were obtained when using 4 wt % ITX and 1 wt % onium salt. Glass slides of thickness 170 ± 5 μm (Marienfeld GmbH, Germany) were precoated with (3-glycidyloxypropyl) trimethoxysilane (Sigma Aldrich) in order to achieve better adhesion between the polymer and the glass surface, and the resins were dropcast onto them. Then, a glass slide was mounted on a three-axis piezo stage (P-562.3CD, Physik Instrumente (PI), Germany) with a bidirectional positioning accuracy of 2/2/4 nm (*x*/*y*/*z*) and a 200 μm travel range in each direction. The writing speed was 2 μm/s. The ITX was excited by laser pulses of 780 nm (82 MHz repetition rate, 110 fs, FFS-tSHG, Toptica, Germany). TAD was performed via a continuous wave (CW) 660 nm laser (Opus, Laser Quantum, Germany). The power of the excitation beam was controlled by an acousto-optical modulator (MT110-A1.5-IR, AA Opto Electronic, France), while the power of the TAD beam was adjusted directly by the laser. All powers quantified in this paper were powers entering the back aperture of the objective lens (60×, NA 1.49, oil immersion, Olympus, Japan). While writing, we could observe a small point (possibly fluorescence from ITX) moving forward in the scan direction. After writing the lines, the samples were developed by rinsing with 5 to 10 drops of ethanol until the sample was visibly clean. For depletion experiments, two ordinary foci were confocalized, and the heights of the written lines were determined with an AFM (DI, CP-II, Digital Instruments, USA). In the case of STED-inspired TAD lithography, a 2π phase spiral (RPC Photonics, Rochester, NY, USA) was used to create a donut-shaped focus, placed around the 780 nm excitation focus. Linewidths were determined with a Zeiss 1540XB SEM after evaporating approx. 10 nm of gold.

## Experimental Results

In a first experiment, we wrote lines on a glass substrate using different compositions of EPOX, ITX, and onium salts and varied the two-photon excitation power. The best results for our purposes were obtained using 4 wt % ITX and 1 wt % onium salts (other compositions are shown in Supporting Information, Figures S1 and S2). Excitation powers were 3.2–3.4 mW for both ITX/onium salt compositions. Without a depletion laser, the lines were typically 180 (185) nm high and 350 (500) nm wide when using Ar_3_S:SbF_6_ (Ph_2_I:PF_6_) as an onium salt, respectively. It is well-known in MPL that the heights of lines written directly on a glass substrate can be well below the axial size of the point spread function.^41^ Compared to linewidths down to 100 nm achieved with high numerical aperture lens MPL lithography in acrylates,^2,3^ 350 to 500 nm linewidths might sound quite large; however, we note that in the case of photochemically initiated cationic polymerization, linewidths are typically four to five times as thick as comparable linewidths achieved with (meth)acrylates.^19,20^

**Figure 2:**
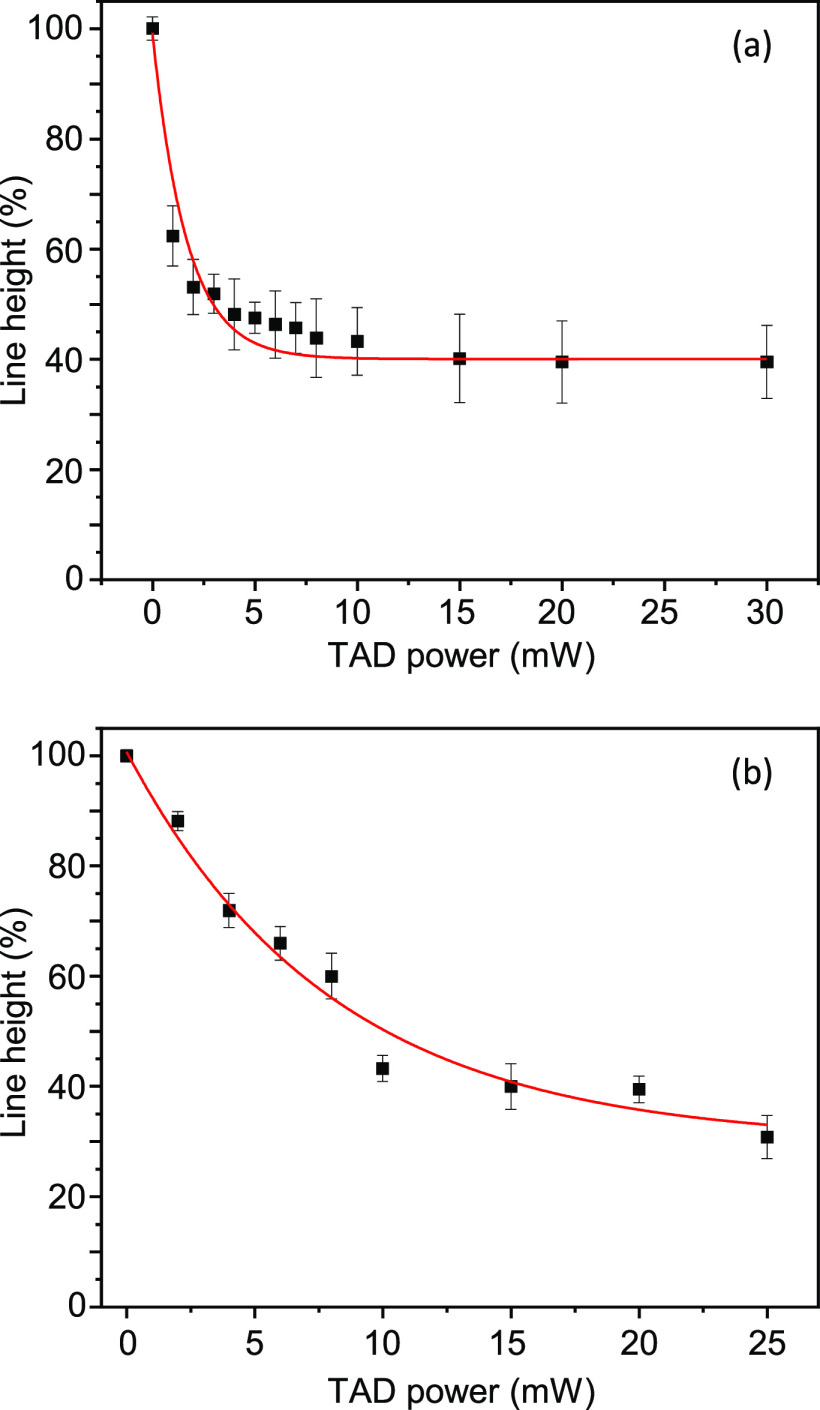
Suppression of polymerization: line height (measured with AFM) as a function of transient absorption depletion (TAD) power. The excitation power was 3.3 mW. Starter compositions: (a) 4 wt % ITX, 1 wt % Ar_3_S:SbF_6_ and (b) 4 wt % ITX, 1 wt % Ph_2_I:PF_6_. The red lines are fitted single-exponential decays. The residuals are 40 and 30% in the case of Ar_3_S:SbF_6_ and Ph_2_I:PF_6_, respectively. The 1/*e* exponential decay powers *P*_sat_ are 1.7 ± 0.2 and 8.1 ± 1.6 mW in the case of Ar_3_S:SbF_6_ and Ph_2_I:PF_6_, respectively.

Starting from line heights of around 180 nm (measured with AFM), we gradually increased the depleting TAD power, confocalized with an ordinary PSF on top of the MPL excitation focus. Figure 2 shows the relative reduction in line height as a function of TAD power for both resists, ITX/Ar_3_S:SbF_6_ and ITX/Ph_2_I:PF_6_ in panels a and b, respectively. One sees a roughly exponential decrease in line height with increasing TAD power. The red lines are exponentially decaying numerical fits to the data. In both cases, the lines do not totally disappear, but residuals of 40 and 30% remain in the cases of ITX/Ar_3_S:SbF_6_ and ITX/Ph_2_I:PF_6_, respectively. Higher TAD powers than 30 or 25 mW, respectively, were not applied in order to not destroy the sample due to microexplosions caused by excessive 660 nm optical power, which occur irrespective of a simultaneous 780 nm excitation. It is quite clear that there are apparently two species of polymerizations, one that can be depleted by 660 nm light and another one that cannot and is responsible for the residual line height. We will discuss that in more detail in the Discussion section. The species, which can be depleted, showed threshold depletion powers (defined as the power where 1/*e* of the line height was reached) of *P*_sat_ = 1.7 ± 0.2 and 8.1 ± 1.6 mW in the case of Ar_3_S:SbF_6_ and Ph_2_I:PF_6_, respectively. These powers play a similar role in STED-inspired lithography to that played by the so-called saturation powers *P*_sat_ in STED microscopy, which are the depletion powers where the fluorescence has dropped down to 1/*e* of its original value without depletion. It has been shown by Hell and Westphal^42^ that the minimally achievable resolution in STED microscopy as a function of the applied depletion power *P*_depl_ is given by
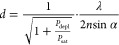
1whereby λ is the vacuum wavelength of light, *n* is the refractive index of the immersion medium, and α is the half-opening angle of the objective lens. Comparing now ITX/Ar_3_S:SbF_6_ with ITX/Ph_2_I:PF_6_ as a starter system for EPOX polymerization, we can conclude that ITX/Ar_3_S:SbF_6_ bears the advantage of having the lower saturation power, while ITX/Ph_2_I:PF_6_ has the advantage of having the lower amount of undepletable background. In the discussion, we will argue that both effects are inter-related.

The best possible depletion of the line height, ideally down to the complete vanishing of lines, as typically seen in acrylate-based STED-inspired lithography,^43^ is desirable in order to achieve the narrowest feature sizes. Unfortunately, this is apparently not the case in both EPOX systems that we used in this study. Nevertheless, this does not preclude the use of these systems. In TAD lithography, a comparatively long-lived transient state is used to optically switch off the polymerization. This is somewhat related to the so-called RESOLFT (reversible saturable optical fluorescence transitions) microscopy,^6^ where in diffraction-unlimited fluorescence microscopy, a long-lived state is optically manipulated. Indeed, in the first reports about RESOLFT microscopy, it was also not possible to totally switch off fluorescence with a depleting beam. Very similar to our case (c.f. Figure 2), about 40% of fluorescence could not be switched off, but still, a subdiffractional resolution was achieved.^6^ Therefore, we turned to STED-inspired photoinhibition lithography.

**Figure 3:**
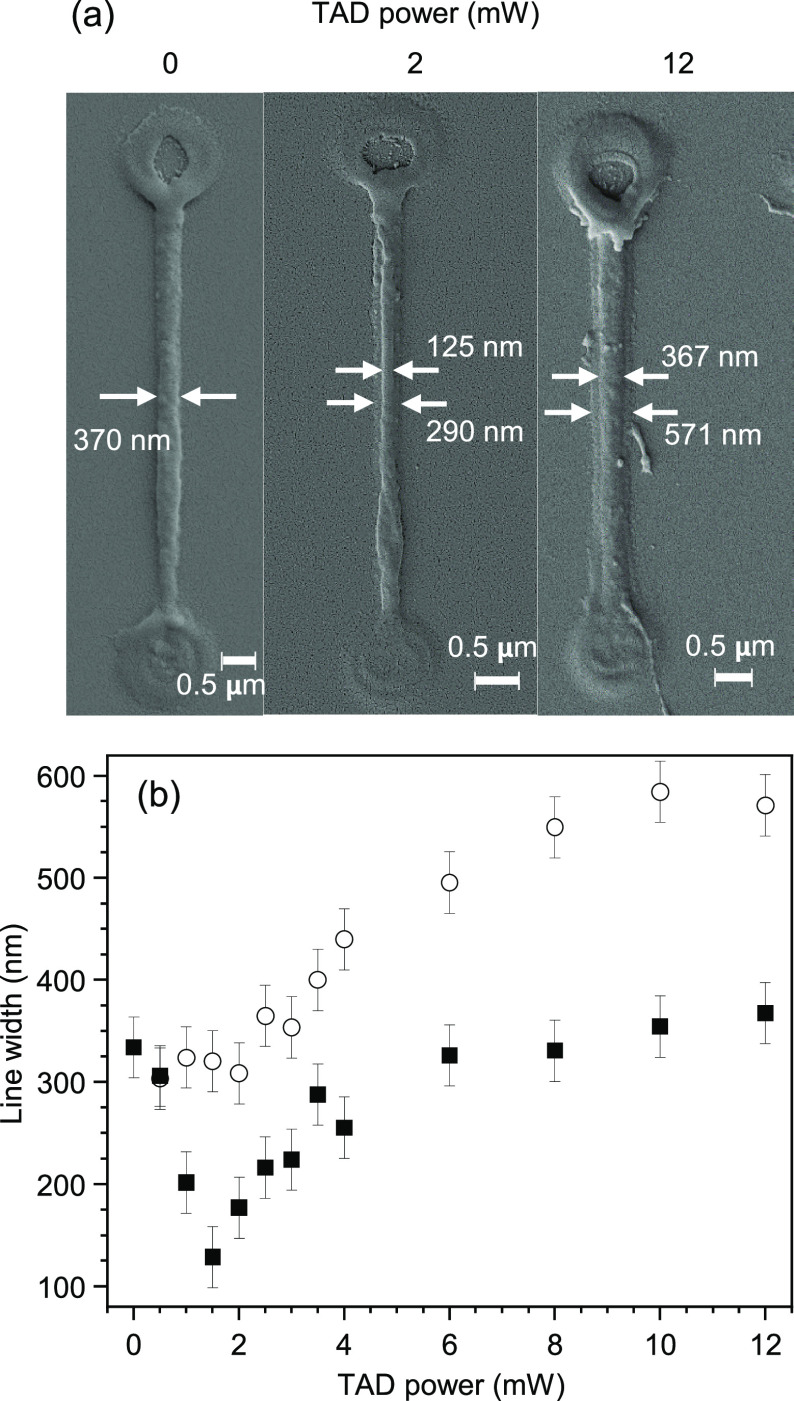
(a) Lines written with 3.2 mW of 780 nm excitation power and different TAD powers as indicated, using 4 wt % ITX and 1 wt % Ar_3_S:SbF_6_. The pure MPL (0 mW TAD) linewidth is 370 nm. Applying 2 mW of TAD, the central line narrows by 66% down to 125 nm, but a faint pedestal of 290 nm width is visible. In the case of 12 mW, the central line is as wide as the line without TAD, and the pedestal widens to 571 nm. (b) Widths of the central lines (filled squares) and the pedestals (open circles). Data points in (b) are averages of up to 4 independent experiments.

In order to test the minimally achievable linewidths, we switched from an ordinarily shaped PSF of the 660 nm depleting beam to a donut-shaped PSF by inserting a 2π phase mask. After developing the lines, they were overcoated with 10 nm of gold and imaged in an SEM. Figure 3a shows some lines with ITX/Ar_3_S:SbF_6_ as the sensitizer/initiator system. The excitation power was 3.2 mW, and the TAD powers were as indicated. The large blobs at the beginning and ending of each line are anchoring points, written with a high excitation power of 6 mW. This fixates the lines and avoids washing them off during development. The frequently observed central hole in the middle of the anchors might be due to some ablation. When no TAD power was applied (i.e., pure MPL lithography), a linewidth of 370 nm was achieved in this example. This is a typical linewidth for MPL lithography of epoxides, where minimal linewidths are typically 3 to 5 times wider than typically achievable widths of MPL-written acrylate lines.^19,20^ Applying a 2 mW TAD power, the linewidth shrank down to 125 nm in this specific case, which is a 2/3 improvement compared to the linewidth without TAD. However, on closer inspection, one recognizes a pedestal underneath the central thin line, which has a width of 290 nm. When 12 mW TAD was applied, the width of the central line was nearly as wide as the linewidth without TAD and the width of the pedestal grew up to 571 nm. We wrote lines for several other TAD powers and repeated the whole series four times. SEM images of all four runs of measurements and tables with the measured linewidths are given in the Supporting Information (Figure S4 and Tables S1 and S2). Figure 3b shows the linewidths, averaged over the four runs, as a function of TAD power. The full squares show the widths of the central lines, and the open circles show the widths of the pedestals.

When the TAD power was increased up to 1.5 mW, the width of the central lines shrank by about two-thirds down to 130 nm on average. Although this result is well above the routinely achievable linewidths of less than 50 nm in free radical-initiated, acrylate-based STED-inspired photoinhibition lithography, it is still a major achievement because this is, to the best of our knowledge, the first time that STED-inspired lithography showed a marked effect on linewidth in cationically initiated polymerization. Given the 14 years advancement of free radical-initiated STED-inspired lithography, this is a remarkable first result for STED-inspired, cationically initiated lithography.

When increasing the TAD power beyond 1.5 mW, the central lines broaden again, approaching the original linewidth from 6 mW TAD power onward. In other words, a STED-inspired line narrowing can only be observed between 0.5 and approx. 5 mW TAD power, with a minimal linewidth at 1.5 mW. In addition, the pedestal widens substantially from approx. 3 mW onward, saturating at an about 600 nm linewidth. We will suggest a reason for the origin of this pedestal in the discussion.

We also tried to write lines with the iodonium salt as a photoinitiator. Unfortunately, the results were not well-repeatable from run to run; specifically, the lines did not stick to the substrate and contained bulges and blobs. Some results are shown in Supporting Information, Figure S5. In the case of some rare, successfully written lines, we found a 0 mW TAD linewidth (pure MPL) of 400 to 600 nm and also observed about 2/3 shrinkage down to 220 nm when about 6 mW of TAD was applied. Beyond that TAD power, the linewidths increased again. Interestingly, the minimal linewidths achieved with Ar_3_S:SbF_6_ and Ph_2_I:PF_6_ were 1.5 and 6 mW, respectively, both powers being close to the saturation intensities *P*_sat_ of 1.7 and 8.1 mW obtained in the depletion experiments (Figure 2). Because of the 4.5 times higher TAD power for minimal linewidths and the overall poor performance of line stability in the case of ITX/Ph_2_I:PF_6_, we conclude that ITX/Ar_3_S:SbF_6_ is the far better photoinitiator system for STED-inspired photoinhibition lithography of EPOX.

## Theoretical Estimations

**Figure 4:**
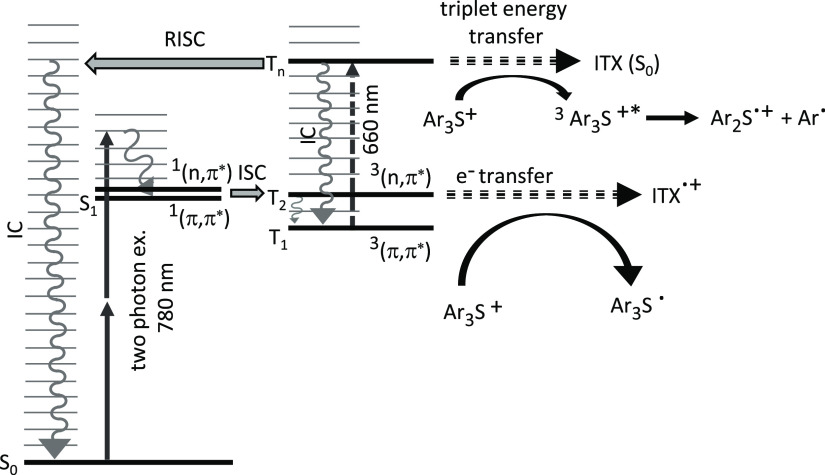
Jabłonski diagram of ITX. After 780 nm two-photon excitation, ITX undergoes intersystem crossing (ISC) to the triplet system, from where it can excite an onium, e.g., Ar_3_S^+^, via electron transfer. If an additional 660 nm light is provided, ITX is further excited to *T_n_*, from where it can either fall back to *T*_1_ via internal conversion (IC) or undergo reverse intersystem crossing (RISC) followed by fast IC down to *S*_0_, or it can transfer its energy to the onium. Further details are described in the text.

We herewith put our results into context with the well-established framework of ITX-sensitized initiation of cationic polymerization. The energy levels of thioxanthones have been studied in great detail before^32−36^ and are summarized in Figure 4, the left part of which represents a Jabłonski diagram showing singlet and triplet levels relevant for our discussion. After two-photon excitation,^19^ the ITX will temporarily be excited into a higher vibrational level of ^1^(π,π*), from where it quickly transfers via intersystem crossing (ISC) to ^3^(n,π*) followed by internal conversion (IC) down to ^3^(π,π*).^34^ Alternatively, it first undergoes IC within the singlet system to ^1^(n,π*) followed by ISC to a higher vibrational level of ^3^(π,π*) followed by IC within the ^3^(π,π*).^36^ Both pathways obey El-Sayed’s rule,^44^ which means that ISC is very effective and the ITX ends up in the triplet system after some picoseconds. It depends on the polarity of the monomer which of the two routes prevails.^34,36^ As both of them equally end up in the ^3^(π,π*) state, it is futile and beyond the scope of this paper to discuss this in detail.

Once in the *T*_1_ = ^3^(π,π*) state, the initiation of a cationic polymerization proceeds via a thermal repopulation of the *T*_2_ = ^3^(n,π*) state followed by a rapid transfer of an electron from the sensitizing ITX toward the aryl-onium cation.^45,46^ This process is shown in the lower right part of Figure 4 for the case of Ar_3_S^+^, but the sensitization of Ph_2_I^+^ via electron transfer works analogously. Although ITX is commonly called the “sensitizer”, this is actually misleading because it is predominantly the resulting ITX^·^^+^ radical cation, which itself induces cationic polymerization. As the ITX needs to be thermally activated within the triplet system prior to electron transfer, the overall process is rather slow.^30,45^ This gives time for the second, deactivating continuous wave (CW) laser beam of 660 nm wavelength to excite the ITX from *T*_1_ = ^3^(π,π*) into a higher triplet level *T_n_* via an optically allowed transient-state absorption.^45^ From the *T_n_* level, the molecule has three options to proceed: The first and most effective pathway is IC within the triplet system back down to the *T*_1_. If we assume that IC within the singlet and within the triplet systems is of similar probability, the rate of intratriplet IC is approximately γ_TIC_ = (0.4 ps)^−1^.^36^ The second possibility is a reverse intersystem crossing (RISC in Figure 4) followed by quick IC within the singlet system. This will bring the ITX back to the ground state and will lead to an effective transient (here: triplet)-state absorption depletion (TAD) process, which is also the core process for subdiffractional STED-inspired lithography of acrylates using ITX as a two-photon radical starter.^24−26,39,40^ In the following, we assume that the rates of RISC and ISC are similar. The published timescales for ISC actually vary between 4 and 10 ps.^34,36,40^ For the further discussion, we take the average and assume γ_RISC_ = (7 ps)^−1^, keeping in mind that this is probably the least well-known parameter in the following considerations. Nevertheless, it is for sure an order of magnitude less effective than intratriplet IC.

Even slower is a third possibility: An ITX molecule in the *T_n_* state has sufficient energy to excite the onium via triplet energy transfer.^23,30^ In this sense, ITX acts as a “true” sensitizer of the Ar_3_S^+^ (or the Ph_2_I^+^). During this process, the ITX turns from the *T_n_* down to the *S*_0_, and simultaneously, the Ar_3_S^+^ is excited to its triplet state, from where it decays into an Ar^·^ cation and an Ar_2_S^·^^+^ radical cation, which initiates polymerization. As the ITX turns from a triplet to a singlet and the Ar_3_S^+^ from a singlet to a triplet, this energy transfer is spin-conserving and hence allowed. However, we assume that the rate of this third process is on a typical scale of energy transfers, which means γ_ET_ = (500 ps)^−1^, two orders of magnitude lower than RISC and three orders of magnitude lower than intratriplet IC.

For the time being, we will neglect the slow process of energy transfer and concentrate on the decay of the *T_n_* on the branching ratio between RISC and intratriplet IC. In order to render TAD effective, RISC needs to outperform the thermal activation from ^3^(π,π*) to ^3^(n,π*) followed by an electron transfer (c.f. Figure 4). This initiation process can indeed be depleted by TAD, if the molecule is continuously being excited from the *T*_1_ to the *T_n_* followed by intratriplet IC back down to *T*_1_, thereby effectively cycling the molecule between the *T*_1_ and the *T_n_*. Each time that the molecule is in *T_n_*, there is a finite probability for RISC to escape this cycle and therefore to escape the triplet system. The quantum efficiency to escape per one cycle is η_esc_ = γ_RISC_/(γ_RISC_ + γ_TIC_) = 5.4%.

In STED microscopy, a decisive parameter is the so-called saturation power *P*_sat_ of the STED beam, which is defined as the power where the remaining (not depleted) fluorescence has dropped down to 1/*e* of its original value.^42^ Transferring this terminology to STED-inspired lithography, *P*_sat_ would be the power where the photoinitiation via electron transfer is depleted via TAD down to 1/*e* of its original value. In order to calculate that, we first need to know the quantum efficiency of electron transfer without TAD. If an ITX is in its *T*_1_ = ^3^(π,π*) state, it can undergo several reactions: First, it might decay back down to *S*_0_. This rate has been found to be γ_0_ = 0.1 μs^–1^.^45^ Next, it might be quenched by electron transfer to an EPOX monomer. This leads to a reduction of an EPOX monomer; however, such a reduced EPOX cannot start a polymerization reaction. Hence, this is a loss process.^45^ Given a concentration of the EPOX monomer (which is the monomer and the solvent at the same time) as [EPOX] = 4.41 M and taking the rate of quenching by the monomer as *k*_EPOX_ = 6.0 M^–1^ μs^–1^,^45^ we end up with a rate of quenching by the monomers of γ_EPOX_ = *k*_EPOX_·[EPOX] = 26.5 μs^–1^. Next, as we do not work oxygen-free, the *T*_1_ state of the ITX will be quenched by oxygen. The corresponding rate can be estimated by γ_ox_ = 4 μs^–1^.^45^ Finally, there is the desired triplet quenching via electron transfer to the onium. In the case of Ar_3_S^+^, the molar quenching rate is found to be *k*_Ar_3_S_ = 210 M^–1^ μs^–1^.^30^ With a concentration of Ar_3_S^+^ units of [Ar_3_S^+^] = 10.73 mM, we end up with γ_Ar_3_S_ = 2.25 μs^–1^. Without TAD, the total quenching rate is therefore γ_noTAD_ = γ_0_ + γ_EPOX_ + γ_Ox_ + γ_Ar3S_ = 32.8 μs^–1^. The inverse of it, τ_T_ = 30 ns, is actually the lifetime of the ITX triplet state within the resist, and the quantum efficiency for an electron transfer is η_elT, noTAD_ = γ_Ar3S_/γ_noTAD_ = 6.9%. We also note in passing that working oxygen-free would increase this efficiency only to 7.8%, so it is not worth the effort to exclude the oxygen, as usual when working with cationic polymerization.

We now can turn to theoretically estimate *P*_sat_, which we define as the TAD power, where the polymerization, which is initiated by electron transfer, is reduced down to 1/*e*. This means that the quantum efficiency for an electron transfer with the depleting beam switched on needs to decrease down to 1/*e*, or
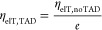
2whereby η_elT, TAD_ = γ_Ar3S_/(γ_noTAD_ + γ_TAD_), which leads to the condition
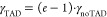
3From the above discussion about the branching ratio between RISC and IC from *T_n_* back down to *T*_1_, it follows that γ_TAD_ = γ_TT_·η_esc_, whereby the triplet–triplet absorption rate γ_TT_ can be estimated from published oscillator strengths. Rai-Constapel et al. showed that, within the triplet system, the *T*_1_→*T*_8_ transition is by far the dominating one with an oscillator strength of *f* = 0.33,^35^ in accordance with Harke et al., who calculated *f* = 0.26 for the *T*_1_→*T*_8_ transition.^26^ Without specifying the final *T_n_* state, Mundt et al. found *f* = 0.28.^36^ We therefore assume that *f* ≈ 0.3 is a reasonable average. From this, we can deduce (see the Supporting Information) an absorption cross section in the peak of the transient absorption spectrum of σ_TT,max_ = 7.83 × 10^–17^ cm^2^. This peak is at about 630 nm, with a 60 nm full-width at half-maximum.^26^ Therefore, we assume the triplet–triplet absorption cross section at 660 nm (the wavelength of the TAD laser) to be σ_TT_ = 3.9 × 10^–17^ cm^2^.

The diffraction-limited diameter of the ordinarily shaped PSF of the TAD beam used for the depletion measurements is 220 nm, and therefore, the depletion area can be estimated by *A* = 3.80 × 10^–10^ cm^2^. The photon flux density in the focus is given by
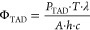
4with *P*_TAD_ being the TAD power going into the objective lens, *T* = 0.9 being the transmission of the objective lens at λ = 660 nm (specified by the manufacturer), *h* being Planck’s constant, and *c* is the speed of light. With the equality γ_TT_ = Φ_TAD_·σ_TT_ and eqs 3 and 4, we arrive at the following formula for the saturation power, where the polymerization should have dropped down to 1/*e* of its original value:
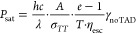
5

Inserting all numerical values, we receive *P*_sat_ = 3.4 mW. This is twice the experimental value of 1.7 mW (Figure 2a); however, it is remarkably close to it, considering how many parameters went into this estimation. We stress that all values were reasonably based upon published data (either experimental or theoretical) of the dynamics of ITX or closely related thioxanthones. The largest uncertainty probably lies in the rate of RISC. This was assumed to be similar to the rate of ISC, for which published data vary from (10 ps)^−1^ to (4 ps)^−1^.^31,33,35^ Furthermore, it is reasonable to assume that due to Fermi’s golden rule, the rate of RISC is larger than that of ISC because of the higher density of the final RISC states within the highly excited singlet system compared to the low number of triplet levels in the range of *T*_1_ and *T*_2_ available for ISC. This would increase the escape efficiency η_esc_ and consequently reduce the theoretically estimated value for *P*_sat_. Finally, we would like to stress that our above considerations on the TAD process in ITX as a photosensitizer for cationic polymerization also shed light on the TAD process in the case of ITX as a one-component type II starter for radical polymerization.^40^ Instead of an electron transfer to an onium, hydrogen abstraction within the same or from another ITX molecule takes place.^37^ To the best of our knowledge, it has so far not been considered in detail why RISC can actually outperform the intratriplet IC back to the *T*_1_ followed by hydrogen abstraction. In a similar way, the radical starters 7-diethylamino-3-thenoylcoumarin (DETC),^24^ Michler’s ethyl ketone,^43^ or some diketones^47^ are depletable by TAD, and a similar consideration as outlined above might be applied to explain the intramolecular dynamics that renders TAD effective in the case of radical polymerization lithography using these starters.

## Discussion

When comparing the depletion efficiencies of Ar_3_S:SbF_6_ and Ph_2_I:PF_6_, it appears that Ar_3_S:SbF_6_ is more effective than Ph_2_I:PF_6_. This is clearly seen when comparing the saturation powers in Figure 2, which are 1.7 ± 0.2 and 8.1 ± 1.6 mW in the case of Ar_3_S:SbF_6_ and Ph_2_I:PF_6_, respectively. This is almost a factor of 5 difference. However, this can be explained if one has a closer look at the electron transfer process. It is allowed if
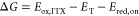
6is negative.^46^ Here, *E*_ox,ITX_ is the ground-state oxidation potential of ITX, *E*_T_ is the triplet energy of ITX above the *S*_0_, and *E*_red,on_ is the reduction potential of the onium. While Δ*G* < 0 holds for both Ar_3_S^+^ and Ph_2_I^+^, it is well-documented that the reduction potential of Ph_2_I^+^ is some 100 mV less negative than that of Ar_3_S^+^, and therefore, Δ*G* is more negative in the case of Ph_2_I^+^ compared to Ar_3_S^+^ as an electron acceptor.^30,46,48^ This has the consequence that electron transfer from a triplet ITX to Ph_2_I^+^ is up to a factor of 10 more feasible than to Ar_3_S^+^.^30^ This shortens the *T*_1_ lifetime (or increases γ_noTAD_ and hence *P*_sat_ in eq 5). Consequently, more TAD power is needed so that RISC can compete with (and finally outperform) electron transfer. This theoretical consideration nicely fits to our experimental result that *P*_sat_ is larger in the case of Ph_2_I:PF_6_ compared to Ar_3_S:SbF_6_, see Figure 2.

Next, we give a plausible explanation for the undepletable residuum in the height of the lines in Figure 2 and, simultaneously, for the pedestal that appears when more than 1 mW of 660 nm light is applied and broadens upon a further increase of the 660 nm TAD power (Figure 3b, open circles). Obviously, this polymerization cannot be stopped by 660 nm and even grows in width with increasing 660 nm power. Hence, the initiation of polymerization should be of a different nature than the so far discussed route of electron transfer followed by the formation of an ITX^·^^+^ radical cation. Furthermore, the pedestal also appears when an ordinarily shaped (instead of a donut-shaped) PSF of 660 nm light is applied, see Supporting Information, Figure S3.

Indeed, there is an alternative possibility, which is shown in Figure 4 on the upper right. Starting from the *T*_1_, the absorption of a 660 nm photon excites the ITX to a higher triplet state *T_n_*, possibly the *T*_8_. From there, it has sufficient energy that a triplet energy transfer from the ITX toward the onium can take place, leaving the onium in an excited triplet state, which promptly dissociates into an aryl-onium radical cation and an aryl cation.^21,45^ This is the natural way how onium salts initiate cationic polymerizations if excited directly in the UV region. Most important, this polymerization path is inaccessible to further manipulation with light; hence, this is a dark reaction path, and TAD cannot be used for depletion. Further, as an energy transfer has a typical rate on the order of γ_ET_ = (500 ps)^−1^, this process is rather sluggish compared to the electron transfer route and hence only appears at high powers of 660 nm light. Nevertheless, it competes with the photoinitiation via the electron transfer process. Hence, the faster the electron transfer is, the less effective is the triplet energy transfer route. Above, we noted that Δ*G* is more negative for Ph_2_I^+^ compared to Ar_3_S^+^, and hence, *P*_sat_ is smaller in the case of Ar_3_S:SbF_6_ compared to Ph_2_I:PF_6_. The same reason, the more negative Δ*G*, disfavors the triplet energy transfer route with respect to the optically depletable electron transfer route, and hence, the residuum is larger in the case of the Ar_3_S:SbF_6_ compared to Ph_2_I:PF_6_. Consequently, the smaller *P*_sat_ and the larger residuum in the exponential fit in the case of Ar_3_S:SbF_6_ (Figure 2a) as compared to Ph_2_I:PF_6_ (Figure 2b) can both be traced back to the more negative Δ*G* in the case of Ph_2_I^+^.

While the broad pedestals that start to appear at around 2 mW have now been associated to being caused by energy transfer, we assign the thin (and depletable) central lines to being started by electron transfer. This route can effectively be depleted by TAD. Hence, the linewidths narrow from 350 down to 130 nm when increasing the TAD power up to 1.5 mW. However, beyond this TAD power, they start to broaden again until they reach the original width of approx. 350 nm. This means that TAD becomes less effective with increasing power and eventually ceases to have any influence on the linewidth. We argue that local heating might be responsible for this effect. The 660 nm beam pumps quite some energy into the system.^49^ First, it cycles the ITX molecule within the triplet system between *T*_1_ and T_*n*_, whereby the intratriplet IC releases thermal energy. If, eventually, RISC occurs, further thermal energy is liberated due to the IC within the singlet system. This local increase of thermal energy can have two consequences. First, the electron transfer from the triplet system to the onium is thermally activated via the *T*_2_ state. Thus, increased thermal energy renders the electron transfer more efficient, and hence, TAD becomes less efficient in relation to the energy transfer. The second reason might be that a local heating yields a local decrease in viscosity, and therefore, the ITX^·^^+^ and the initially activated monomers become more mobile, which increases the area, where polymerization can occur.^50^ The latter effect might also contribute to the increasing widening of the pedestal with increasing 660 nm power. However, we note that the line height as a function of TAD power seems to converge to a constant residuum and does not increase again, at least not in the investigated power range, which was technically limited by the onset of microexplosions. Further research is needed to clarify this.

## Conclusions

We have shown, for the first time, that STED-inspired photoinhibition lithography can be achieved with cationic photoinitiated epoxides. This is achieved by using ITX and a sulfonium salt as the photoinitiating species. After two-photon excitation, ITX can be depleted via transient-state absorption (TAD), and the electron transfer from the triplet ITX to the sulfonium can be prevented. This way, no ITX^·^^+^ radical cations form and polymerization is suppressed in the outer rim of the excitation point spread function when a 660 nm TAD laser is applied in a donut shape around the central two-photon excitation focus. Linewidths of 130 nm could be achieved, which are only one-third of the linewidths achievable with pure two-photon lithography. Different from acrylate-based STED-inspired photoinhibition lithography, the polymerization could not be suppressed completely. We attribute this to the fact that the ITX, excited to a high triplet level by the 660 nm TAD beam, can sensitize the sulfonium via triplet energy transfer. Subsequently, the sulfonium dissociates and initiates epoxide polymerization, which we assign for being responsible for the undepletable pedestal underneath the thin depletable lines. Further, the repeated intratriplet internal conversion and the intrasinglet IC both dump thermal energy into the focal area, which increases the mobility of the starters and hence might contribute to the observed broadening of both the pedestal and the central line. Further, the additional thermal energy may lead to an accelerated electron transfer and therefore renders TAD less efficient.

This picture conclusively describes most of our experimental findings, although we admit that further research is necessary to complete the picture. However, this work represents, to the best of our knowledge, the first STED-inspired photopolymerization lithography beyond radically polymerizing resins. We addressed several critical points to render TAD efficient, such as the branching ratio of reverse intersystem crossing and intratriplet internal conversion, or the quantum efficiency of TAD, competing with a thermally activated electron transfer, triplet quenching by the monomers and, in the case of higher TAD powers, with triplet energy transfer. Future work has to optimize these branching ratios in order to make TAD more efficient and to increase the writing speed. Currently, we are only able to write supported lines, while experiments trying to write higher dimensional structures such as suspended lines, or lower-dimensional structures such as individual voxels, failed so far. Also, postbaking steps could not improve the outcome of our experiments with EPOX. We are, however, very optimistic that three-dimensional nanoscale structures will be realized in the near future, possibly with other epoxide monomers that allow for better cross-linking and pre- and postbaking or postexposure routines. We are confident that our experimental findings and theoretical considerations substantially help to guide the way toward efficient STED-inspired subdiffractional lithography using cationic polymerization.

## Supporting Information Available

TAD efficiencies for various ITX and onium concentrations, development of the pedestal as a function of TAD power using an ordinary 660 nm PSF, SEM images of four runs of photoinhibition experiments and evaluation of linewidths, TAD photoinhibition lines with Ph_2_I:PF_6_ as the initiator, and calculation of σ_TT_ from oscillator strength *f* (PDF)
