# Supplementary material for: STED-Inspired Cationic Photoinhibition Lithography
Source: J Phys Chem C Nanomater Interfaces. 2023 Sep 7;127(37):18736–44. doi: 10.1021/acs.jpcc.3c04394 (PMC10518867; doi:10.1021/acs.jpcc.3c04394)
Supplement: Supplementary file 1 — jp3c04394_si_001.pdf [file jp3c04394_si_001.pdf]

STED-Inspired Cationic Photoinhibition Lithography

Sourav Islam[1], Marco Sangermano[2], Thomas A. Klar[1,*]

[1]Institute of Applied Physics, Johannes Kepler University Linz, 4040 Linz, Austria
[2]Department of Applied Science and Technology, Politecnico Di Torino, 10124 Torino, Italy

* thomas.klar@jku.at

Supporting Information

1. TAD efficiency measurements

In the main manuscript, we show the achieved line heights (measured with AFM) as a function of increasing TAD power in the case of 4 wt.% ITX and 1 wt.% onium salt. Here, we show the results of similar measurements with other concentrations in Figure S1 for ITX/Ar_3S:SbF_6 and in Figure S2 for ITX/Ph_2I:PF_6 as the starter systems. In case of ITX/Ar_3S:SbF_6, the composition 4 wt.% ITX, 1 wt.% Ar_3S:SbF_6 clearly shows the fastest depletion and the lowest residuum of undepletable line height. In case of Ph_2S:PF_6, the composition 4 wt.% ITX, 1 wt.% Ph_2S:PF_6, shows the smallest residuum, as well, however, some other compositions show very similar results.

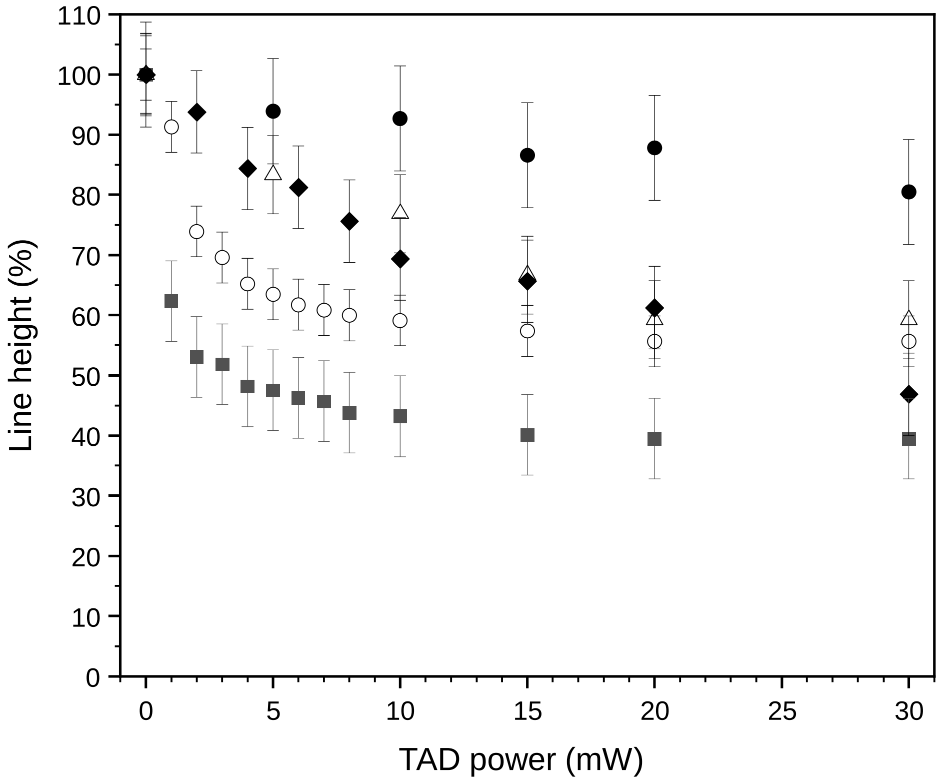

Figure S1. Suppression of EPOX polymerization with 5 different $Ar_3S:SbF_6$ containing starter compositions, as a function of Transient Absorption Depletion (TAD) power. At high TAD powers, the line heights approach a horizontal residuum.
■) 4 wt.% ITX, 1 wt.% $Ar_3S:SbF_6$, residuum 40%
○) 2 wt.% ITX, 1 wt.% $Ar_3S:SbF_6$, residuum 55%
△) 4 wt.% ITX, 0.5 wt.% $Ar_3S:SbF_6$, residuum 60%
●) 2 wt.% ITX, 0.5 wt.% $Ar_3S:SbF_6$, residuum 80%
◆) 4 wt.% ITX, 2 wt.% $Ar_3S:SbF_6$, residuum 46%.

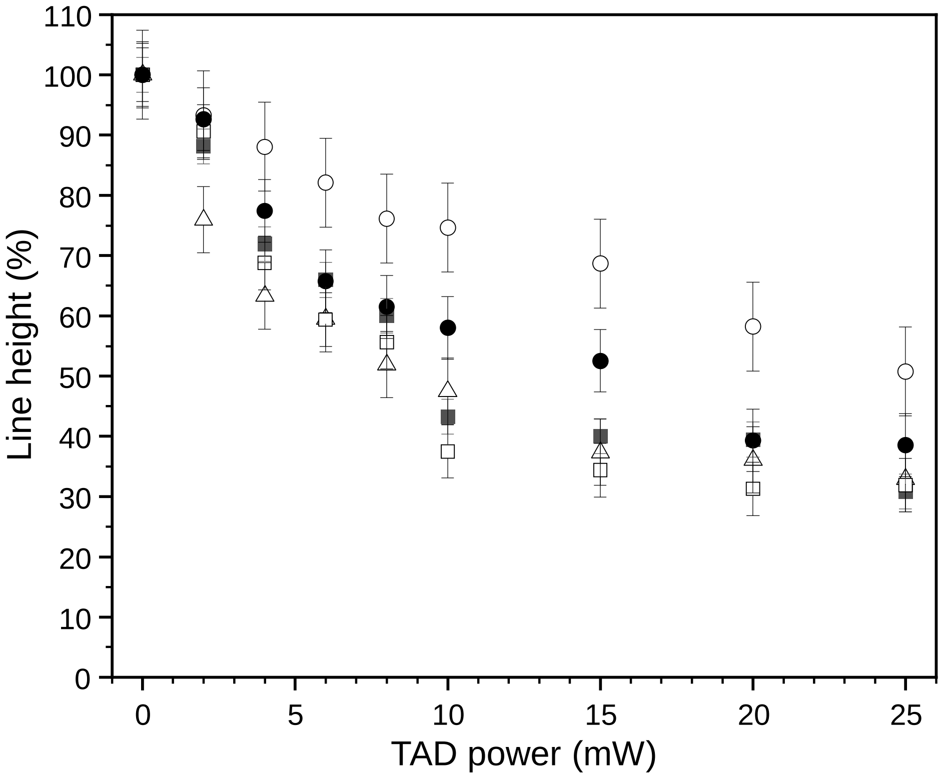

Figure S2. Suppression of EPOX polymerization with 5 different $Ph_2S{:}PF_6$ containing starter compositions, as a function of Transient Absorption Depletion (TAD) power. At high TAD powers, the line heights approach a horizontal residuum.

■) 4 wt.% ITX, 1 wt.% $Ph_2S{:}PF_6$, residuum 30%

○) 2 wt.% ITX, 1 wt.% $Ph_2S{:}PF_6$ residuum 50%,

△) 4 wt.% ITX, 0.5 wt.% $Ph_2S{:}PF_6$, residuum 32%

●) 2 wt.% ITX, 0.5 wt.% $Ph_2S{:}PF_6$, residuum 38%

□) 6 wt.% ITX, 0.5 wt.% $Ph_2S{:}PF_6$, residuum 32%.

2. Development of pedestal with ordinarily shaped TAD PSF

In the main text, we show in Figure 3 how the width of the central line first shrinks and then widens again with increasing TAD power, and how a broad pedestal evolves as a function of increasing TAD power (Figure 3). In these measurements, a donut-shaped TAD PSF was used and the starter system was 4 wt.% ITX and 1 wt.% $Ar_3S:SbF_6$.

For the starter system 2 wt.% ITX and 1 wt.% $Ph_2I:PF_6$, we wrote a series of lines with an ordinarily shaped TAD PSF, confocalized with the excitation PSF and show the results in Figure S3. The excitation power was 3.4 mW. While the width of the central line stays at around 450 nm, the width of the pedestal increases up to 900 nm for 15 mW of TAD power.

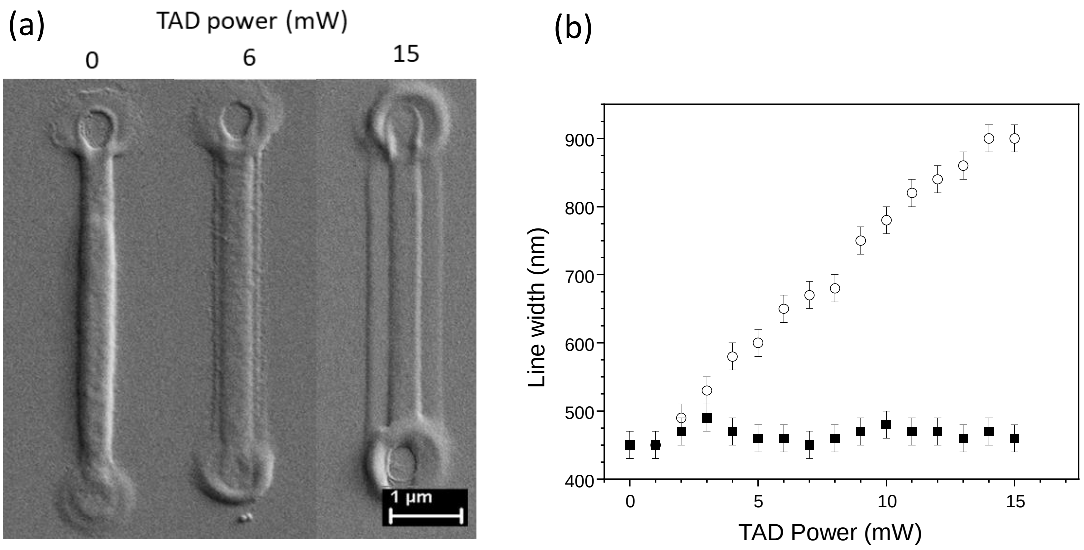

Figure S3. Lines written with confocalized ordinary point spread functions of 780 nm, 3.4 mW and 660 nm TAD. The starter comprised 2 wt.% ITX and 1 wt.% $Ph_2S:PF_6$. (a) Three examples of lines with 0, 6, and 15 mW of TAD power applied. The width of the central line stays essentially the same, while the pedestal widens. (b) Width of the central line (full squares) and the pedestal (open circles) as a function of TAD power.

3. Several batches of linewidths with donut-shaped TAD beam

Figure S4 shows the four different TAD-power series used to determine the average widths of the pedestal and the central line that lead to Figure 3 in the main text. The excitation power is given to the left of each SEM image and the applied TAD power is given above each line.- The starter was 4 wt.% ITX and 1 wt.% $Ar_3S:SbF_6$.

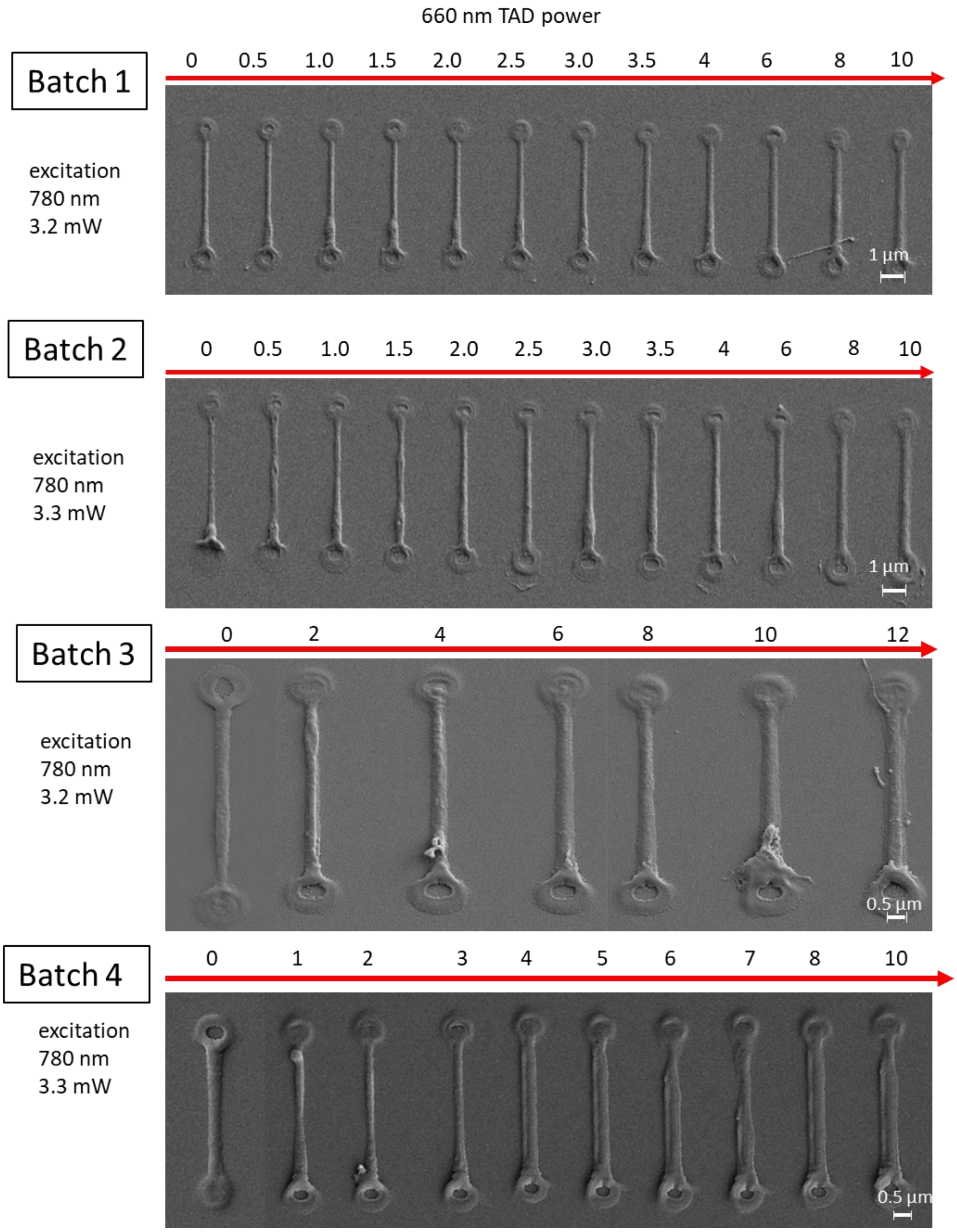

Figure S4. Lines written with 3.2 or 3.3 mW of excitation power and increasing 660 nm TAD power. The TAD-PSF was donut shaped.

All lines shown in Figure S4 were individually imaged with the SEM using a larger magnification and for each line the width of the central line and the width of the pedestal (if visible) was evaluated at four different positions and averaged. The following tables contain these averaged linewidths and in the last column the average of the (up to) four batches. Not each TAD power was applied in each batch, so sometimes the average is taken from less than

four lines. The averages in the last column are the data for the graph presented in Figure 3b in the main manuscript.

TAD power in mW	batch 1 (nm)	batch 2 (nm)	batch 3 (nm)	batch 4 (nm)	average inner width (nm)
0	286	287	370	393	334
0,5	271	341			306
1	132	273		200	202
1,5	125	133			129
2	147	264	125	172	178
2,5	287	145			216
3	312	150		210	224
3,5	270	305			288
4	369	169	180	303	255
6	415	336	200	353	326
8	360	352	210	401	331
10	373	400	280	405	364
12			367		367

Table S1: Inner linewidths for different TAD powers from up to 4 lines from the batches shown in Figure S4.

TAD power in mW	batch 1 (nm)	batch 2 (nm)	batch 3 (nm)	batch 4 (nm)	average outer width (nm)
0	n/a	n/a	n/a	n/a	n/a
0,5	303				303
1	319			329	324
1,5	321				321
2	337	348	290	259	309
2,5		365			365
3		363		344	354
3,5		400			400
4		427	310	582	440
6		456	390	640	495
8	538	521	450	690	550
10	539	6005	480	718	584
12			571		571

Table S2: Outer width of the pedestals for different TAD powers from up to 4 lines from the batches shown in Figure S4.

4. TAD photoinhibition lines with $Ph_2I:PF_6$ as onium starter

While we were able to repeatedly write thin lines with TAD photoinhibition lithography using $Ar_3S:SbF_6$ as onium salt, the results using $Ph_2I:PF_6$ were far less reliable. Many lines detached from the samples and those which stuck were frequently distorted. Some examples are shown in Figre S5 with excitation and TAD powers as indicated. Two examples are enlarged, one with 0 mW TAD (i.e. pure MPL) and one with 6 mW TAD.

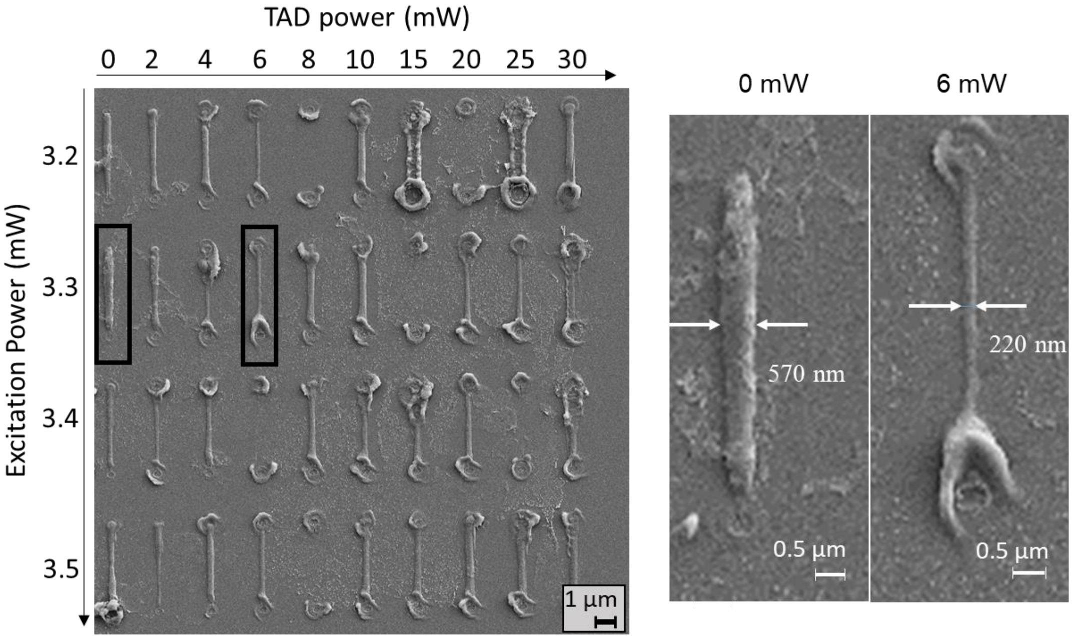

Figure S5. Lines written with 3.2 to 3.5 mW of excitation power and increasing 660 nm TAD power as indicated. The starter system was 4 wt% ITX and 1 wt.% $Ph_2I:PF_6$. Two lines highlighted with black boxes are enlarged on the right hand side.

5. Triplet-triplet absorption cross-section

Several papers have been published where the oscillator strengths of the transient triplet-triplet absorption where calculated for ITX or very similar thioxanthones. Rai-Constapel et al. showed that for thioxanthone the $T_1 \rightarrow T_8$ transition is by far the dominating one with an oscillator strength of $f = 0.33$,[1] while the other TT absorptions show negligible oscillator strength. Without specifying the specific TT transition, Mundt et al. found $f = 0.28$.[2] Harke et al. calculated the TT absorptions for ITX as a single-component type II initiator of an acrylate polymerization and found that again the $T_1 \rightarrow T_8$ is by far the dominating one with $f = 0.26$.[3] We therefore assume that $f \approx 0.3$ is a reasonable average.

The oscillator strength and the absorption cross section $\sigma(\lambda)$ are related in the following way:

$$f = \frac{4 \cdot m \cdot c^2 \cdot \varepsilon_0 \cdot \varepsilon_r}{e^2} \int_0^\infty \frac{\sigma(\lambda)}{\lambda^2} d\lambda \qquad \text{(S1)}$$

m is the electron mass, *c* the vacuum speed of light, ε_0 the vacuum permittivity, and *e* the charge of an electron, and we assume a relative permittivity of EPOX of $\varepsilon_r = 2.24$ at optical frequencies. If we assume that the TT absorption spectrum is approximately Gauss-shaped, the integral can be replaced in good approximation by $\sigma_{TT,\max} \cdot \Delta\lambda / \lambda_{\max}^2$. This leads to the following relation for the peak cross section of the transient TT absorption:

$$\sigma_{\mathrm{TT,max}} = \frac{\lambda_{\max}^2}{\Delta\lambda} \cdot \frac{e^2}{4mc^2\varepsilon_0\varepsilon_r} \cdot f = 7.83 \cdot 10^{-17}\,\mathrm{cm}^2$$

According to Harke et al., the transient TT absorption peaks at $\lambda_{\max} = 630\,\mathrm{nm}$ and obeys a full width half maximum of $\Delta\lambda = 60\,\mathrm{nm}$.[3] This corresponds well with results of the Fouassier-group, who published that the TT absorption of ITX peaks at 605 nm in methanol,[4] and they note that the spectra of thioxanthones bathocromically shift by approx. 30 nm in nonpolar solvents.[5] Therefore we assume that the triplet-triplet absorption cross section at 660 nm is $\sigma_{\mathrm{TT}} = 3.9 \cdot 10^{-17}\,\mathrm{cm}^2$.

References

1. Rai-Constapel, V.; Villnow, T.; Rysek, G.; Gilch, P.; Marian, C. M., Chimeric Behavior of Excited Thioxanthone in Protic Solvents: II. Theory. *Journal of Physical Chemistry A* **2014,** *118*, 11708-11717.
2. Mundt, R.; Villnow, T.; Torres Ziegenbein, C.; Gilch, P.; Marian, C. M.; Rai-Constapel, V., Thioxanthone in apolar solvents: ultrafast internal conversion precedes fast intersystem crossing. *Physical Chemistry Chemical Physics* **2016,** *18*, 6637-6647.
3. Harke, B.; Dallari, W.; Grancini, G.; Fazzi, D.; Brandi, F.; Petrozza, A.; Diaspro, A., Polymerization Inhibition by Triplet State Absorption for Nanoscale Lithography. *Adv. Mater.* **2013,** *25*, 904-909.
4. Manivannan, G.; Fouassier, J. P., Primary Process in the Photosensitized Polymerization of Cationic Monomers. *J. Polym. Sci., Part A: Polym. Chem.* **1991,** *29*, 1113-1124.
5. Kunze, A.; Müller, U.; Tittes, K.; Fouassier, J. P.; Morlet-Savary, F., Triplet quenching by onium salts in polar and nonpolar solvents. *J. Photochem. Photobiol. A* **1997,** *110* (2), 115-122.
